# An improved neutral landscape model for recreating real landscapes and generating landscape series for spatial ecological simulations

**DOI:** 10.1002/ece3.2145

**Published:** 2016-05-09

**Authors:** Maarten J. van Strien, Cornelis T. J. Slager, Bauke de Vries, Adrienne Grêt‐Regamey

**Affiliations:** ^1^ Planning of Landscape and Urban Systems (PLUS) Institute for Spatial and Landscape Planning ETH Zurich Stefano‐Franscini‐Platz 5 CH‐8093 Zürich Switzerland; ^2^ Flood Risk Management Deltares P.O. Box 177 2600 MH Delft The Netherlands; ^3^ Information Systems group Department of the Built Environment Eindhoven University of Technology P.O. Box 513 5600 MB Eindhoven The Netherlands

**Keywords:** Landscape metrics, landscape visualizations, spatial optimization algorithms, spatial or time series

## Abstract

Many studies have assessed the effect of landscape patterns on spatial ecological processes by simulating these processes in computer‐generated landscapes with varying composition and configuration. To generate such landscapes, various neutral landscape models have been developed. However, the limited set of landscape‐level pattern variables included in these models is often inadequate to generate landscapes that reflect real landscapes. In order to achieve more flexibility and variability in the generated landscapes patterns, a more complete set of class‐ and patch‐level pattern variables should be implemented in these models. These enhancements have been implemented in Landscape Generator (LG), which is a software that uses optimization algorithms to generate landscapes that match user‐defined target values. Developed for participatory spatial planning at small scale, we enhanced the usability of LG and demonstrated how it can be used for larger scale ecological studies. First, we used LG to recreate landscape patterns from a real landscape (i.e., a mountainous region in Switzerland). Second, we generated landscape series with incrementally changing pattern variables, which could be used in ecological simulation studies. We found that LG was able to recreate landscape patterns that approximate those of real landscapes. Furthermore, we successfully generated landscape series that would not have been possible with traditional neutral landscape models. LG is a promising novel approach for generating neutral landscapes and enables testing of new hypotheses regarding the influence of landscape patterns on ecological processes. LG is freely available online.

## Introduction

The spatial arrangement and composition of anthropogenic land uses and natural habitats can have a significant impact on a range of ecological processes (Turner 1989), such as plant dispersal (e.g., Higgins et al. 2003), metapopulation viability (e.g., With et al. 2006), population abundance (e.g., Flather and Bevers 2002), species richness (e.g., Steiner and Köhler 2003), habitat connectivity (e.g., Mimet et al. 2013), or genetic differentiation between populations (e.g., Bruggeman et al. 2010; Van Strien et al. 2015). Instead of using real landscapes, many of these studies have simulated these ecological processes in computer‐generated landscapes (Gardner and Urban 2007; Wang and Malanson 2008; but see, for instance, Mimet et al. 2013). In order to determine the influence of a specific landscape composition or configuration variable on an ecological process, series of landscapes can be generated in which the concerned variable is incrementally changed while other variables are kept constant (Saura and Martínez‐Millán 2000; Neel et al. 2004; Cambui et al. 2015). Compared to using real landscapes, computer‐generated landscapes are less restricted in the range of possible landscape patterns and capable of capturing only those aspects of landscape patterns that are relevant to the study objectives (Wang and Malanson 2008). Here, we use the term “landscape pattern” to refer to landscapes with certain configuration and composition of land‐use patches.

Computer‐generated landscapes can originate from “process‐based” models that simulate a process, which in turn shapes the landscape. For instance, Pe'er et al. (2013) created a process‐based landscape generator that simulates land‐use patterns of agriculture and forest patches that could result from roads traversing the landscape. Similarly, Gaucherel et al. (2012) model the effect of agricultural production systems on configuration and composition of patchy agricultural areas. Although such process‐based models are highly suitable to assess how certain processes influence landscape patterns, they are not designed to generate landscapes in which these processes are not the main drivers of the composition or configuration. In process‐based models, the input parameters give direction to the process, which, as an output, produces a landscape. For instance, from the list of model parameters in Pe'er et al. (2013), the number of roads and the size of agricultural fields were the main parameters influencing the composition and configuration of the generated landscapes. Thus, only with a thorough understanding of how the different input parameters influence the resulting landscape, can a specific landscape pattern be generated.

In contrast to process‐based models, neutral landscape models “produce an expected pattern in the absence of specific landscape processes” (Gardner et al. 1987; p. 19). Although originally developed to test whether observed landscape patterns can be resulting from random spatial processes (Gardner et al. 1987), more recently, neutral landscape models have frequently been used to test hypotheses about the influence of landscape patterns on spatial ecological processes (Saura and Martínez‐Millán 2000; Gardner and Urban 2007; Cambui et al. 2015). Percolation maps, a first generation of neutral landscapes, had the number of land‐cover categories *n* and their probability of occurrence *P* as only input settings, on the basis of which raster cells were randomly assigned to the *n* categories (e.g., Gardner et al. 1987). Such percolation maps were used by Saura and Martínez‐Millán (2000) to generate landscapes with varying patch sizes and proportions of land‐use classes. Making use of midpoint displacement algorithms, an additional input parameter *H*, specifying the correlation between raster cells, was then implemented to vary the fragmentedness of the simulated landscape from highly fragmented (*H *=* *0) to highly aggregated (*H* = 1; Gardner 1999; Gardner and Urban 2007; Cambui et al. 2015). Additional control over the final pattern of the simulated landscape can be achieved with the “minimum mapped unit”, which controls the scale or degree of detail of a simulated landscape (Saura 2003), or the neighborhood criterion, which indicates which neighboring raster cells are used to assess the spatial relationships in the landscape (e.g., four or eight neighbors). Variations to the above methods are, for instance, hierarchical or curdled maps (Gardner 1999), vector‐based maps with irregular patch shapes (Le Ber et al. 2009), or random combinations of Gaussian functions (Wiegand et al. 1999). Recently, Etherington et al. (2015) implemented most of the above methods into the software package NLMpy.

Despite the frequent use of the methods described above, “neutral landscape models have difficulty in capturing all the pattern characteristics of a real landscape by approximating the landscape and class‐level metric values” (Li et al. 2004; p. 146). Discrepancies usually remain between landscape metrics calculated from real landscapes and those from landscapes simulated to represent these real landscapes (e.g., Gardner and Urban 2007). A reason for this discrepancy is that most input variables for neutral landscape models are landscape‐level parameters (e.g., *n*,* H*, minimum mapped unit and neighborhood criterion), meaning that they apply to all land‐use categories in the landscape. In addition, some landscape models do not accommodate landscapes with more than two land‐use classes (e.g., Pe'er et al. 2013; Remmel and Fortin 2013; Cambui et al. 2015). In real landscapes, however, there are often several land‐use classes and configurations may differ between classes and patches (Li et al. 2004). To simulate such landscapes, neutral landscape models should thus have the capability to generate landscapes with several land‐use categories and include class‐ and patch‐level parameters (i.e., parameters that characterize the configuration of individual land‐use classes or of single patches, respectively; McGarigal et al. 2012).

A further difficulty in the reproduction of real landscapes with neutral landscape models is caused by the fact that some input parameters of neutral landscape models are not easy to calculate from real landscapes. For instance, “real landscapes have no value of H” (Neel et al. 2004) and it is thus difficult to determine what value of H should be used to reproduce a specific landscape pattern. Additionally, nonlinear relationships between input parameters and variables used to evaluate landscape patterns (i.e., landscape metrics; Riitters et al. 1995; Cushman et al. 2008; McGarigal et al. 2012) make it difficult to predict what landscape pattern will result from certain input settings (Li et al. 2005). There exists a broad array of landscape metrics that focus on various aspects of configuration, such as the shape complexity, core area, contrast, aggregation, subdivision, and isolation (Cushman et al. 2008; McGarigal et al. 2012; Wang et al. 2014). Many of these configuration metrics are calculated from a relatively small number of basic class‐ and patch‐level metrics, including the number of patches, the area, extent, and edge length of patches, the distance between patches and the adjacency of land‐use classes in the landscape (Riitters et al. 1995; Cushman et al. 2008; McGarigal et al. 2012). Including these basic metrics as input parameters of neutral landscape models would thus improve their ability to capture the patterns characteristic of real landscapes. In summary, in order for neutral landscape models to more accurately reproduce the patterns found in real landscapes, they should ideally be able to generate landscapes with several land‐use classes using a set of easily calculable class‐ and patch‐level landscape metrics as input parameters; an idea recently followed by Slager and de Vries (2013).

The neutral landscape model “Landscape Generator” (LG) developed by Slager and de Vries (2013) allows users to specify many of the above mentioned class‐ and patch‐level landscape metrics to control the pattern of the generated landscape. LG has been developed for participatory spatial planning and has been demonstrated on the planning of, for instance, residential areas at a relatively small scale (i.e., parcel level; Slager 2011; Slager and de Vries 2013). Here, we demonstrate how LG can also be used at larger scales to generate landscapes for ecological simulation studies. Furthermore, in earlier versions of LG, it was difficult for users to change the input settings and calculations did not utilize the full calculation capacity of a computer. We improved the performance and usability of LG and made the software freely available online. LG can be considered a neutral landscape model, “as it does not include any explanatory process of the resultant spatial patterns” (Saura and Martínez‐Millán 2000; p. 662). However, the patterns generated by LG are strictly speaking not resulting from random spatial processes and therefore cannot be used as null‐models against which the patterns from real landscapes can be tested (i.e., what neutral landscape models were originally used for; Gardner et al. 1987).

In a first analysis, we show that LG is capable of “recreating” patterns derived from a real landscape, which is a frequently used test to demonstrate the suitability of a neutral landscape model (Saura and Martínez‐Millán 2000; Li et al. 2004; Gardner and Urban 2007; Etherington et al. 2015). However, apart from the proportions of certain land‐use classes in the real landscape, these studies usually do not derive any input parameters for their neutral landscape model from the real landscapes (for reasons mentioned above). Instead, they generate a set of landscapes with incrementally changing input settings and assess whether the range of landscape patterns in this set encompasses the range found in real landscapes (e.g., Saura and Martínez‐Millán 2000; Pe'er et al. 2013). The goal in this study is to recreate a specific real landscape. We do this for a range of landscapes with several land‐use classes and a variety of patterns. In a second analysis, we generate two series of landscapes with incrementally changing configuration variables that could potentially be used to study the effect of landscape pattern on spatial ecological processes (Wiegand et al. 1999; Saura and Martínez‐Millán 2000; Flather and Bevers 2002; Steiner and Köhler 2003; Neel et al. 2004; Bruggeman et al. 2010; Cambui et al. 2015). With traditional neutral landscape models, such series can be generated by changing the proportion of habitat (*p*) and the fragmentedness (*H*; e.g., Neel et al. 2004; Wang et al. 2014; Cambui et al. 2015), but here we include additional pattern variables, thus greatly increasing the potential applications of such series. In the first series, we varied the number of patches of a certain land use together with the largest patch size of this land use. Such landscapes could be used in simulation studies addressing, for instance, the long standing question whether it is more beneficial for species conservation to have a single large conservation area or several small ones (e.g., McCarthy et al. 2011). Furthermore, many landscape ecological studies aim to understand how ecological processes are affected by habitat edges (Ries et al. 2004) or the landscape composition and configuration surrounding habitat patches (i.e., matrix; Rodewald 2003; Prevedello and Vieira 2010). To demonstrate how computer‐generated landscapes can potentially be used to study these effects, we generated a second landscape series, in which we varied the edge length of a patch as well as the degree to which two land‐use types were adjacent to one another.

## Methods

### Landscape generator

LG is based on a multiobjective optimization that “searches” for a landscape that best meets the composition and configuration objectives, which are specified by a user‐defined set of class‐ and patch‐level landscape metrics (Slager and de Vries 2013). To reach the objectives, LG iteratively swaps pairs of raster cells and, after every swap, evaluates whether the new landscape pattern is closer to the objectives. For a more detailed description of the optimization algorithm implemented in LG, we refer to Slager (2011) and Slager and de Vries (2013).

To generate a landscape, LG requires a landscape raster file and a list of input settings (Slager and de Vries 2013). The landscape raster can be a random percolation map in which each raster cell is randomly assigned a land‐use category proportionate to the probability of occurrence of each land‐use type. Alternatively, the input raster can be derived from a real landscape, in which case the user can indicate for which raster cells the land use can change and for which cells it is fixed. The input settings of LG are a list of target values of class‐ and patch‐level landscape metrics (Table 1), most of which are also implemented in the frequently used software package FRAGSTATS (McGarigal et al. 2012). Metrics in the current version of LG are calculated with the four‐neighbor criterion. For some of these landscape metrics, the maximum permissible deviation from the target value can be specified. LG optimizes the objective functions sequentially and, therefore, the order of the list of input settings can influence the optimization speed or success.

**Table 1:** Landscape metrics for which target values can be set in Landscape Generator (LG). The abbreviations of the metrics correspond to those used in FRAGSTATS (McGarigal et al. 2012), except for ARBB/PBB, which is not implemented in FRAGSTATS. The landscape metric ECON as implemented in LG is equivalent to the ECON metric in FRAGSTATS if the contrast between two land‐use classes is set to 1. Modified from Slager and de Vries (2013). Note that “number of patches” (NP) is equivalent to “number of instances” (NI) used in Slager and de Vries (2013)

Landscape metric	Abbreviation	Class‐/patch‐level metric
Proportion of landscape	PLAND	Class
Number of patches	NP	Class
Maximum total edge	TE	Class
Patch size	AREA	Patch
Patch maximum perimeter	PERIM	Patch
Patch edge contrast	ECON	Patch
Patch bounding box aspect‐ratio and proportion of bounding box area	ARBB/PBB	Patch

In order to improve the usability and performance of LG, we implemented several new features in the program. In previous versions of LG, users had to specify the input settings within the LG script itself, which was a laborious procedure and required a detailed understanding of the Java programming language. We modified the original Java script of LG, so that input settings can be specified in a separate text file from which LG then reads the input settings. These input text files can be written manually or generated automatically with any programming language (here we used Python), which greatly enhances the usability of LG. Furthermore, previous versions of LG were only capable of generating one landscape at a time. We have adapted LG to be able to generate multiple landscapes simultaneously using all processor cores in a PC, which results in a decreased computation time when generating multiple landscapes. Once a landscape has been generated, LG will also automatically start generating a next landscape until all input text files have been completed. The speed of calculations is an important aspect to consider when simulating landscapes (Saura and Martínez‐Millán 2000). As demonstrated by Slager and de Vries (2013), LG is capable of optimizing 18 objectives in landscapes of 20 × 20 raster cells within 2 min, which is a reasonable calculation time. In this study, we generate larger landscapes (i.e., 50 × 50 raster cells) and also recorded the calculation times. All analyses were run on a desktop computer (CPU: 8‐core Intel Xeon^®^ 3.60 GHz; Memory: 32.0 GB; OS: Windows 7 64‐bit). The modified version of LG together with a manual and example input files has been made available online (www.lg.ethz.ch). LG is programmed in Java and can be used with most operating systems (e.g., Windows, LINUX and Mac OS).

### Recreating landscape patterns

**Figure 1:**
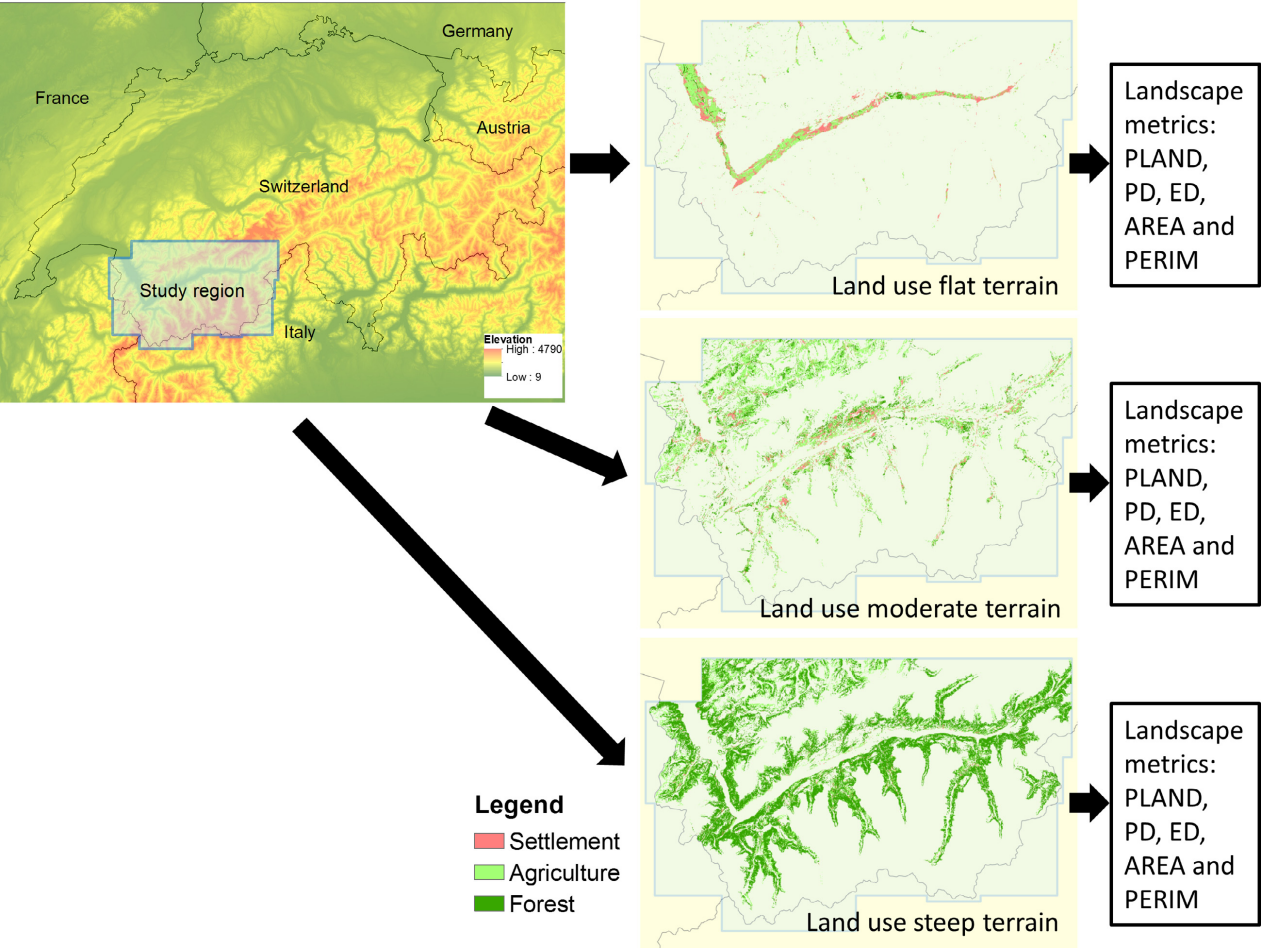
Overview of the process of calculating landscape metrics from a real landscape. The study region is located in the Canton of Valais in the South‐West of Switzerland. Land use in this region was characterized by the aggregated land‐use categories settlement, agriculture, and forest. Land‐use maps were divided based on slope classes: flat = 0–10%; moderate = 11–40%; steep ≥ 41%. For each slope class, the landscape metrics PLAND, PD, ED, AREA, and PERIM (See main text for explanation of the abbreviations; McGarigal et al. 2012) were calculated.

**Figure 2:**
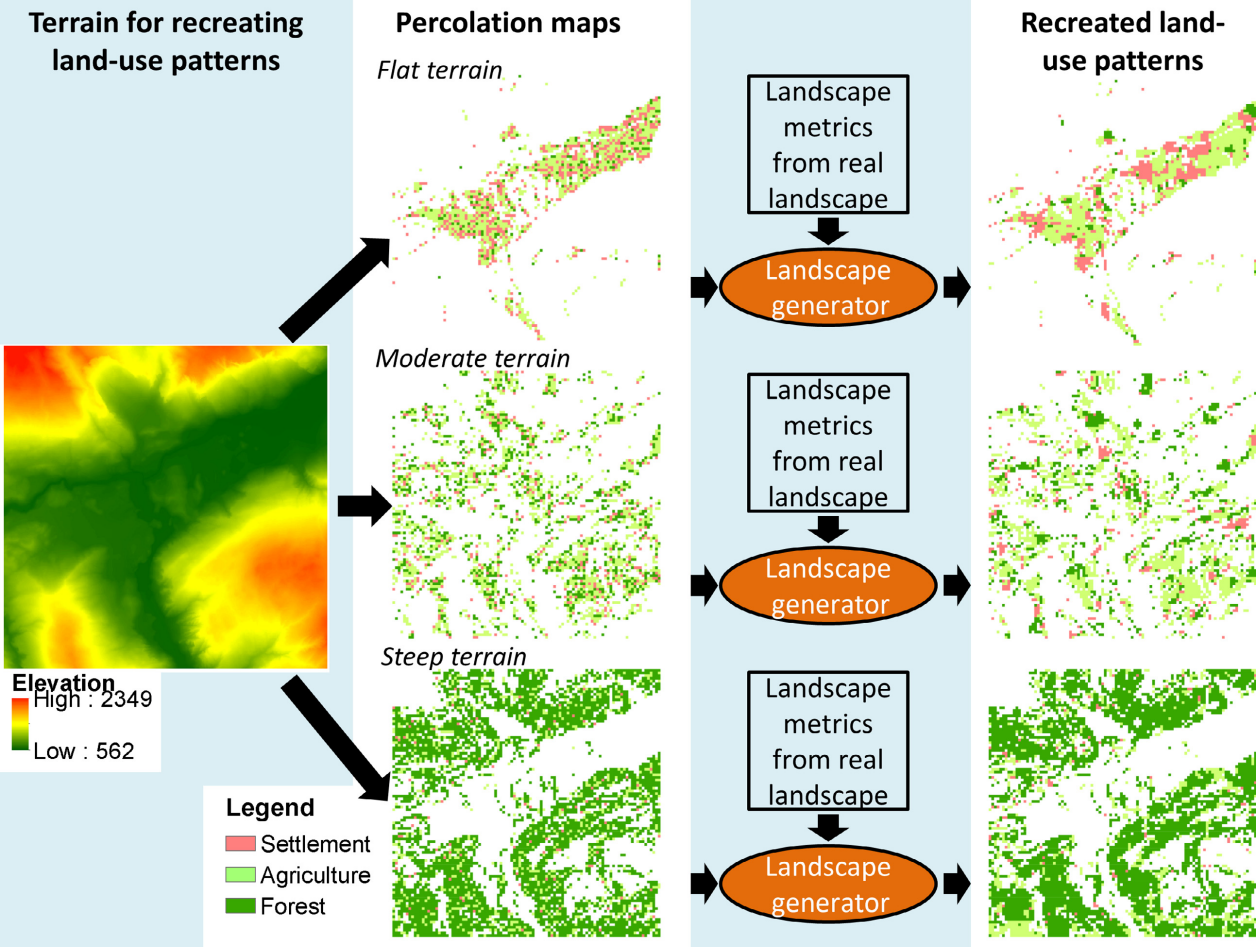
Overview of the analysis steps used to recreate land‐use patterns on a mountainous terrain. This terrain was selected from an area other than our study region (Fig. 1). For the different slope classes (see Fig. 1), we generated percolation maps in which the three different land‐use categories (i.e., settlement, agriculture, and forest) were randomly assigned to raster cells proportionate to the proportion of a certain land‐use in the study region. Together with the landscape metrics calculated from the study region, these percolation maps formed the input to Landscape Generator. The output of Landscape Generator was land‐use patterns that resembled the patterns in the study region.

In a first analysis, we tested how well LG could recreate a landscape pattern derived from a real landscape. The region from which we recreated the landscape pattern is the mountainous Canton of Valais in Switzerland (Fig. 1). For this region, we characterized the landscape pattern with a range of landscape metrics, which were subsequently used as input settings for LG to recreate the original landscape pattern in a different mountain terrain (Figs. 1, 2). We then compared various landscape metrics from the real and the generated landscape to test how accurately the landscape pattern was recreated.

We used land‐use rasters from 2009 (FSO 2013) that covered the whole region (cell size = 1 ha) and that contained four aggregated land‐use categories: settlement, agriculture, forest, and unproductive (i.e., lakes, rivers, rock fields, etc.). We excluded the latter category from further analysis because land‐cover types in this category usually have a high directionality (e.g., rivers run through valleys, landslides are perpendicular to contour lines), which is very challenging to capture when generating landscapes. Land‐use patterns in mountainous regions are very dependent on the slope of the terrain. Therefore, we divided our land‐use rasters into three slope classes: “flat” areas had a percentage slope between 0 and 10%, “moderate” areas had a slope between 11 and 40%, and “steep” areas had slopes of 41% or more. We also excluded areas that were higher than 2000 m in elevation because this is a conservative approximation of the forest line in this region (Szerencsits 2012) and, therefore, the land‐uses forest and settlement hardly occur above this elevation. For the land‐use raster in each slope class (Fig. 1), we calculated landscape metrics with FRAGSTATS (McGarigal et al. 2012). The proportion of landscape (PLAND), the patch density (PD), and the edge density (ED) were the calculated class‐level metrics. As patch‐level metrics, we calculated the area (AREA) and perimeter (PERIM) of each patch (Fig. 1).

In the next step of the analysis, we selected a mountainous terrain of 10 by 10 km (i.e., 100 × 100 raster cells) from a different part of Switzerland (i.e., Canton of Graubünden; Fig. 2), on which the landscape patterns from the Canton of Valais were recreated. From the selected area, we extracted the raster cells for the different slope classes and excluded raster cells that were categorized as unproductive or as higher than 2000 m. Subsequently, we defined the input settings for LG (Appendix S1). The area of each slope class, combined with PD and ED measured in the Canton of Valais (Fig. 1), allowed us to calculate, for a certain land use, the number (NP), and total perimeter of patches (total edge, TE; Appendix S1). Compared to real landscapes, the area of the largest patch is usually smaller in random maps (Gardner and Urban 2007). Therefore, we also calculated the size and perimeter of the three largest patches. From all patches in Canton of Valais, we randomly drew NP patches from which we selected the three largest patches (AREA). To obtain an edge length typical to the patches in Canton of Valais, we predicted the perimeter (PERIM) of these three patches making use of a linear regression (*R* ^2^ > 0.995, *P* < 0.001) between the log(PERIM) and the log(AREA) of all patches in Canton of Valais (Fig. 2; Appendix S1). Only patches with an AREA larger than 2% were considered. For each slope class, we used nine class‐level and 18 patch‐level landscape metrics as input settings of LG (Appendix S1). We refrained from adding more input settings, as preliminary tests showed that optimizations became very slow or failed to converge with more parameters. Since the AREA and patch number of the three largest patches was selected randomly, not every combination of input settings was feasible to simulate. Therefore, we generated input settings 50 times (with different random combinations of AREA and patch number) for each slope class. Since the optimization algorithm implemented in LG contains stochastic components, two landscapes generated with identical input settings will not be entirely identical. In order to get an impression of the variability between generated landscapes, we selected, for each slope class, an input setting that successfully generated a landscape, made 20 copies of the input settings, and generated 20 landscapes with these settings. The landscape raster files on which LG performed the optimizations were simple percolation maps in which raster cells were randomly assigned a land‐use category proportionate to the PLAND (Saura and Martínez‐Millán 2000; Fig. 2).

After the landscapes were generated, we compared the pattern of the real landscape with those of the 20 simulated landscapes by comparing values from class‐level landscape metrics that cover different aspects of landscape patterns. From each of the “highly universal and consistent class‐level landscape structure components” identified by Cushman et al. (2008), we selected landscape metrics (except from the neighborhood similarity metrics as they require a similarity score between land‐use classes): largest patch index (LPI), mean patch shape index (SHAPE_MN), mean Euclidean nearest neighbor distance distribution (ENN_MN), coefficient of variation in Euclidean nearest neighbor distance distribution (ENN_CV), mean edge contrast index (ECON_MN; contrast between settlement and agriculture was set to 1 and all other contrasts to 0), clumpiness index (CLUMPY), interspersion and juxtaposition index (IJI), aggregation index (AI), and normalized landscape shape index (NLSI). Furthermore, we selected PLAND, PD, and ED because these metrics were also used in the LG input settings. Landscape metrics were calculated with FRAGSTATS and for a detailed description of the metrics we refer to the FRAGSTATS user manual (McGarigal et al. 2012).

Although statistical tests for model validation should be treated with extreme caution (Yang et al. 2004), we performed several tests to compare the landscape metrics calculated from the real landscape with those from the 20 repeated landscape simulations. We calculated 95% confidence intervals from the landscape metric values of the 20 simulated landscapes. We then determined whether the value from the real landscape was within the confidence intervals or, in the cases it was not, the deviation between the real value and the upper or lower confidence interval (whichever was smallest; Banks et al. 2005). In order to make the deviation comparable between landscape metrics, we scaled the deviation to the spread (i.e., highest minus lowest values) in the values from the real landscape across the different land‐use categories and slope classes. The existence of a deviation between the metric values from the recreated landscapes and the real landscape does not implicitly imply an unsuitable model (Kleijnen 1995). Therefore, we also assessed, for each landscape metric, the linear relationship between the mean values of the 20 repeated landscapes and the values of the real landscape by grouping the values calculated for the different land‐use categories and slope classes. This relationship can reveal the “accuracy” and “precision” of LG in recreating the individual landscape metrics (Tedeschi 2006). The precision refers to the “amount of scatter about the average mean” and increases with the *r* ^2^ of the linear relationship, while the accuracy is the “systematic deviation from the truth” and increases with the regression's slope and intercept being closer to 1 and 0, respectively (Tedeschi 2006). We thus checked whether the 95% confidence intervals of the intercept contained 0 and whether those of the slope contained 1.

### Producing landscape series

We generated two landscape series with incrementally changing pattern variables (both 50 × 50 raster cells). In the first landscape series (here termed *series 1*), we generated a landscape series with two land uses (i.e., LU1 and LU2), a variable numbers of patches and a variable largest patch size. For LU1 and LU2, PLAND was arbitrarily fixed at 35% and 65%, respectively (Table 2). The number of patches for LU1 was either 3, 9, 15, or 21, while LU2 always consisted of 1 patch. We varied the size of the largest patch by defining four levels of patch size distributions. In level 1, all patches had the same size. In level 4, the area of one of the patches was 90% (i.e., to make the settings not too restrictive) of the area that patch would have if all other patches occupied 1 raster cell. The other two levels (level 2 & 3) were distributed at equal intervals between levels 1 and 4. The maximum deviation of the AREA target value was set to 1 percent point. To ensure quite compact patches for LU1, the total edge (TE) of all patches was defined as 120% of the total edge that would be obtained if each patch was a perfect square (Table 2).

**Table 2:** Input settings for the two landscape series that were generated with Landscape Generator (LG). Landscapes in *series 1* consist of two land‐use (LU) categories and those in *series 2* consist of three land‐use categories. See Table 1 for details on the landscape metrics. A patch of a certain land use is indicated with “p”

Landscape *series 1*		Landscape *series 2*	
Landscape metrics	Target value(s)	Landscape metrics	Target value(s)
PLAND LU1	35	PLAND LU1	25
PLAND LU2	65	PLAND LU2	40
NP LU1	9 (3, 15 or 21)	PLAND LU3	35
NP LU2	1	NP LU1	1
Largest patch level	1, 2, 3, 4^a^	NP LU2	10
TE LU1	267 (=120% of square patches)^b^	PERIM LU1 p0	130, 462, 795 or 1127^c^
AREA LU1 p0	1^b^	TE LU2	800 (=200% of square patch)
AREA LU1 p1	1^b^	ECON between LU1 p0 and LU2	15, 35, 55 or 75
AREA LU1 p2	1^b^		
AREA LU1 p3	1^b^		
AREA LU1 p4	89^b^		
AREA LU1 p5	1^b^		
AREA LU1 p6	1^b^		
AREA LU1 p7	1^b^		
AREA LU1 p8	1^b^		

a Level 1 means all LU1 patches are equal size; level 4 means that the area of the largest LU1 patch is 90% of the value it would have if all other patches had an area of 1 raster cell.

b These values depend of the largest patch level that is chosen for a certain analysis.

c 130 is 130% of a square patch; 1127 is 90% of a long patch that is 1 cell wide.

In the second landscape series (*series 2*), we used three land‐use categories (i.e., LU1, LU2, and LU3) and we varied the maximum edge length of LU1 together with the proportion that LU1 and LU2 were adjacent to one another. For LU1, LU2, and LU3, PLAND was arbitrarily fixed at 25, 40, and 35%, respectively (Table 2). LU1 always consisted of 1 patch of which we varied the maximum edge length (PERIM): 130, 462, 795, and 1127 cell edges. The lowest value (i.e., 130) is 130% of the edge length that the patch would have if it were square. The highest value (i.e., 1127) is 90% of the edge length that would be obtained if the patch were a long line of raster cells one cell wide. LU2 always consisted of 10 patches of which the total edge length was restricted to 800 (=200% of the perimeter if each cell was of equal size and square; Table 2). The proportion of the adjacency between the LU1 patch and the LU2 patches (ECON) was either 15, 35, 55, or 75% (Table 2).

For both *series 1* and *2,* we wrote the 16 target configurations to input setting text files for LG. Random percolation maps were input rasters for LG (see “Recreating landscape patterns”). Both the input settings and input rasters were automatically generated with a Python‐script that made use of ArcGIS 10.2 arcpy‐module (ESRI, Redlands, CA). These Python‐scripts are included in the example data provided with LG.

## Results

### Recreating landscape patterns

**Figure 3:**
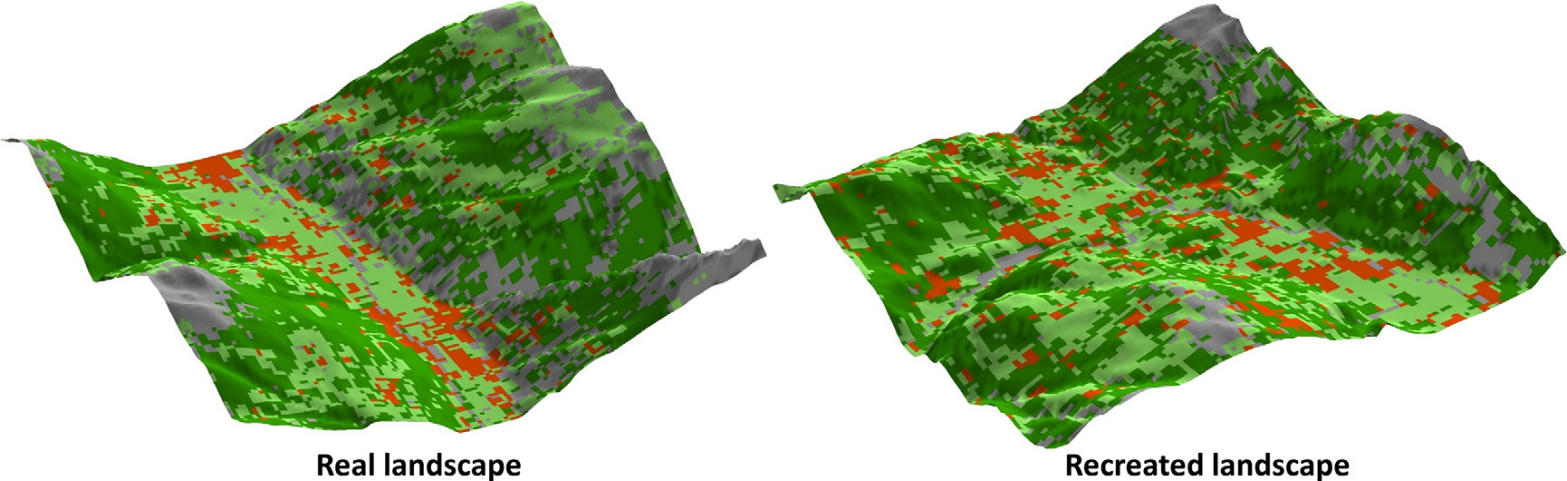
3D perspective views of a part of the real landscape (i.e., Canton of Valais) and the recreated landscape. The landscape patterns of the recreated landscape were simulated with Landscape Generator based on the landscape metrics calculated from the real landscape. The recreated landscape was projected on the terrain that was used to define the input rasters of LG (Fig. 2). Colour codes of the land‐use classes are the same as in Figures 1, 2.

**Figure 4:**
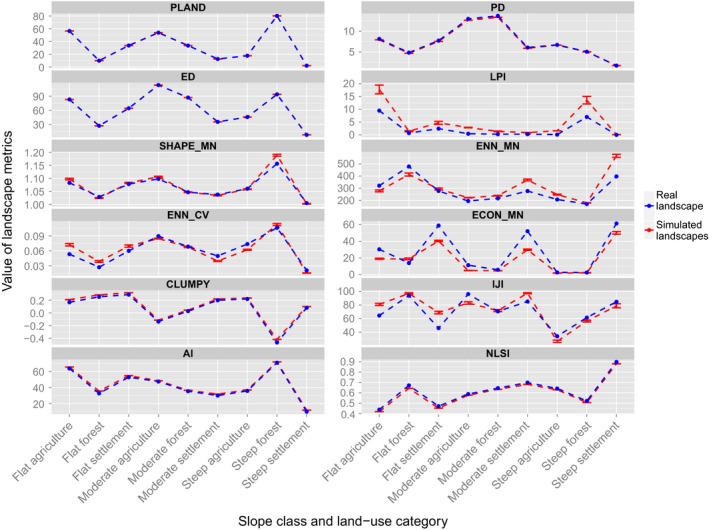
Graphs showing landscape metrics calculated from Canton of Valais (i.e., real landscape) and from landscapes recreated with Landscape Generator. Metrics were calculated for three slope classes (i.e., flat, moderate and steep; Fig. 1) and three land‐use categories (i.e., settlement, agriculture, and forest). For simulated landscapes, the error bars indicate the 95% confidence intervals from the mean value calculated from 20 repeated landscape simulations. The calculated class‐level metrics are: percentage of landscape (PLAND), patch density (PD), edge density (ED), largest patch index (LPI), mean patch shape index (SHAPE_MN), mean Euclidean nearest neighbor distance distribution (ENN_MN), coefficient of variation in Euclidean nearest neighbor distance distribution (ENN_CV), mean edge contrast index (ECON_MN), clumpiness index (CLUMPY), interspersion and juxtaposition index (IJI), aggregation index (AI), and normalized landscape shape index (NLSI).

A visual comparison between the recreated landscape pattern and an equally sized area from Canton of Valais shows that the two landscapes share many land‐use pattern characteristics (Fig. 3). Note that such a visual comparison is difficult, as the terrains underlying both landscapes are different and the depicted real landscape is merely a selection of the landscape that we recreated. Therefore, a comparison of the landscape metrics calculated from the real and recreated landscapes will give a better impression of the similarity between the landscapes. We found that LG successfully recreated the pattern of the real landscape with regard to most, but not all, landscape metrics (Fig. 4; Table 3). The variability between the 20 repeated landscape simulations was low for almost all landscape metrics, except for two land‐use classes (i.e., flat agriculture and steep forest) with the LPI metric (Fig. 4). In general, we found a low mean absolute deviation (≤7.65%) of the recreated landscapes from the real landscape, with only four pattern variables (i.e., LPI, ENN_MN, ECON_MN, and IJI) showing slightly elevated mean deviations (between 11.54 and 16.49%; Table 3).

**Table 3:** Comparisons of real landscapes and landscapes recreated by Landscape Generator (LG). Real and recreated landscapes were compared with 12 different class‐level landscape metrics. For each land use and slope class, we calculated the deviation between the real landscape's metric value and the upper or lower 95% confidence interval (whichever was closer) of 20 repeated landscape simulations. This deviation was scaled to the spread in values in the real landscape. A negative deviation indicates a lower metric value in the recreated landscapes than in the real landscape, while a positive deviation indicates a higher value. A deviation of 0 indicates that the value of the real landscape lies within the confidence intervals of the mean value of the recreated landscapes. The mean of the absolute deviations is also shown. For each landscape metric, a regression was performed between the real landscape and the mean of the 20 recreated landscapes. To determine LG's accuracy, it was assessed whether 1 was within the 95% confidence intervals of the estimated slope and 0 within those of the intercept. The precision of LG was measured with the *r* ^2^. All calculated *r* ^2^ were significant (*P* < 0.01)

Landscape metric	Deviation between metric values from real and recreated landscapes (%)									Mean absolute deviation (%)	Slope = 1	Intercept = 0	*r* ^2^
	Flat agriculture	Flat forest	Flat settlement	Moderate agriculture	Moderate forest	Moderate settlement	Steep agriculture	Steep forest	Steep settlement				
PLAND	0.00	0.07	−0.07	−0.04	0.01	0.03	−0.01	−0.01	0.02	0.03	TRUE	TRUE	1.00
PD	−1.86	−1.36	−1.65	−2.97	−3.04	−1.54	−0.46	−0.40	−0.15	1.49	FALSE	TRUE	1.00
ED	−0.10	−0.04	−0.16	−0.09	−0.07	−0.07	−0.05	−0.03	−0.02	0.07	TRUE	TRUE	1.00
LPI	37.11	3.11	9.42	13.16	5.63	3.39	8.36	28.65	0.41	12.14	FALSE	FALSE	0.99
SHAPE_MN	6.05	−1.48	1.09	3.56	0.00	−0.58	−0.31	15.46	−1.02	3.28	FALSE	FALSE	0.99
ENN_MN	−9.20	−13.76	2.08	6.67	5.04	21.66	9.47	2.16	40.32	12.26	TRUE	TRUE	0.67
ENN_CV	16.85	9.92	7.31	−4.17	0.00	−9.76	−10.72	4.79	−5.29	7.65	TRUE	TRUE	0.86
ECON_MN	−22.16	8.19	−36.76	−12.96	−2.05	−44.44	−1.41	−0.08	−20.33	16.49	TRUE	TRUE	0.92
CLUMPY	5.46	4.06	4.01	3.21	2.15	2.62	1.83	6.62	2.47	3.60	TRUE	FALSE	1.00
IJI	21.05	2.98	29.64	−16.13	0.00	16.24	−7.95	−5.68	−4.22	11.54	TRUE	TRUE	0.71
AI	2.92	4.56	3.21	1.76	1.78	2.84	1.84	1.60	2.98	2.61	TRUE	FALSE	1.00
NLSI	−4.08	−5.93	−4.18	−2.36	−2.30	−3.67	−2.40	−3.25	−3.85	3.56	TRUE	TRUE	1.00

For seven out of 12 landscape metrics, we found slope = 1 and intercept = 0 in regressions between the real and recreated landscape metric values (Table 3), indicating high accuracy in recreating these landscape patterns. Lower accuracy was found for the metrics PD, LPI, SHAPE_MN, CLUMPY, and AI (slope ≠ 1 and/or intercept ≠ 0; Table 3). However, given the extremely high precision of PD, CLUMPY, and AI (i.e., *r* ^2^ = 1.00; Table 3), we assume that, for these metrics, this reduced accuracy is due to rounding effects in the translation of landscape metrics from the real landscape to input settings for LG. Thus, inaccuracies were mainly found for metrics quantifying patch shape complexity (SHAPE_MN) and largest patch dominance (LPI; Cushman et al. 2008).

The precision with which LG recreated certain landscape patterns was generally high (*r* ^2^ ≥ 0.86). Only for IJI and ENN_MN, the lower *r* ^2^ values (0.71 and 0.67, respectively; Table 3) indicated a reduced precision. In this study, IJI and ECON_MN are the two metrics measuring the intermixing of land‐use categories, while ENN_MN quantifies the nearest neighbor distance (McGarigal et al. 2012). Although ECON_MN was accurately and fairly precisely recreated by LG, the mean deviations between the real and recreated landscapes were relatively high for this metric (i.e., 16.49%; Table 3). These discrepancies between real and simulated landscapes indicate that there were differences in the adjacency of land‐use categories and in the distance between patches of a certain land use. These discrepancies may be due to the rather scattered distribution of the areas belonging to a certain slope class in our simulated landscapes (Fig. 2), which could lead to a situation in which patches of a certain land use cannot be placed as close together as in the real landscape. For the same reason, target values of AREA could exceed the area actually available for a certain patch, which could explain why LG could not generate landscapes for all of the 50 repeated input settings. Although edge contrast index (ECON) can be included as input setting for LG, we refrained from using this metric, as pattern optimizations with LG would have become too time consuming. For the successfully generated landscapes, simulations took between 2:45 and 22:00 h to complete on a single processor core.

### Producing landscape series

**Figure 5:**
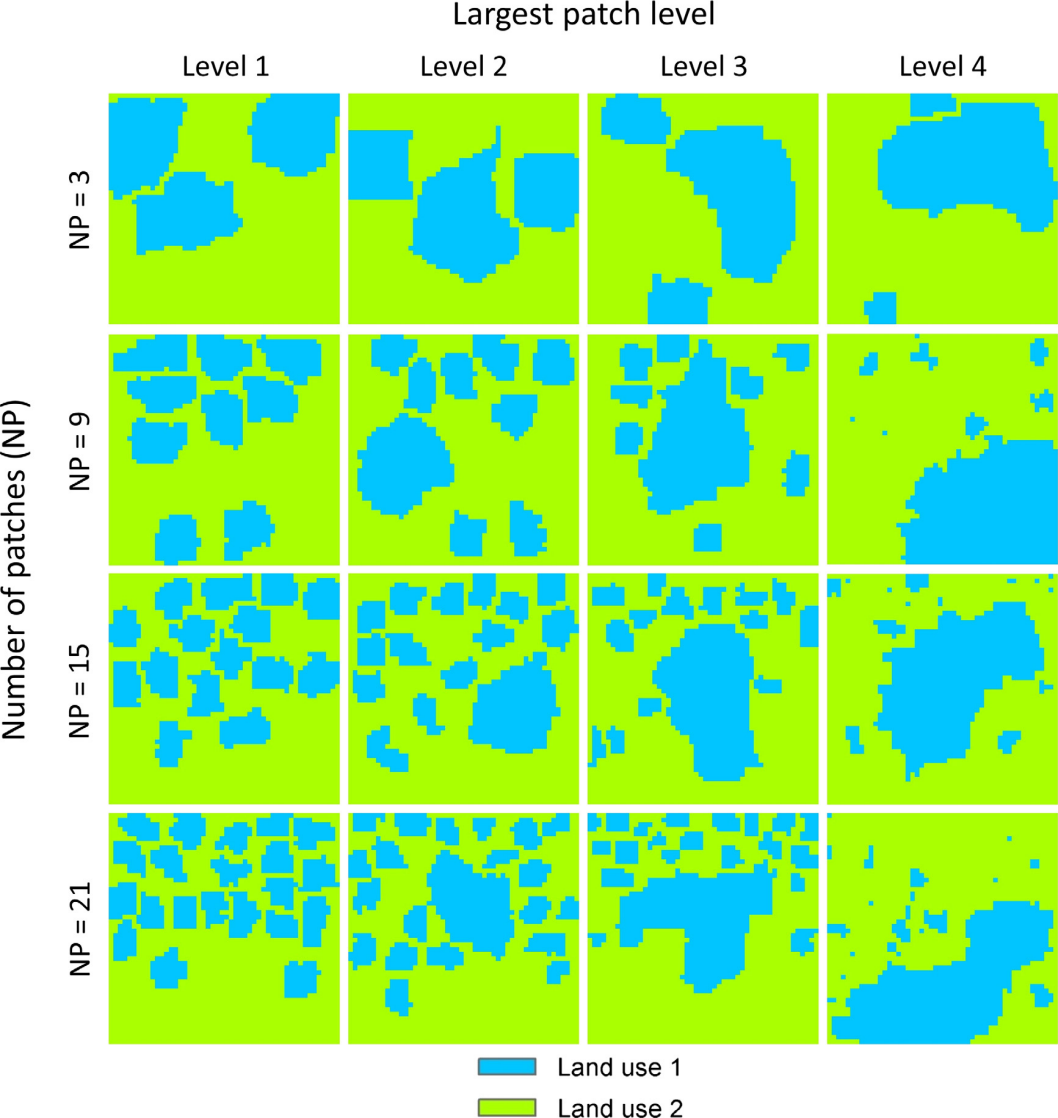
Landscape *series 1* generated by Landscape Generator. We generated a landscape series with two land uses and with variable numbers of patches together with variable largest patch sizes. For a detailed overview of the input settings, see Table 2.

**Figure 6:**
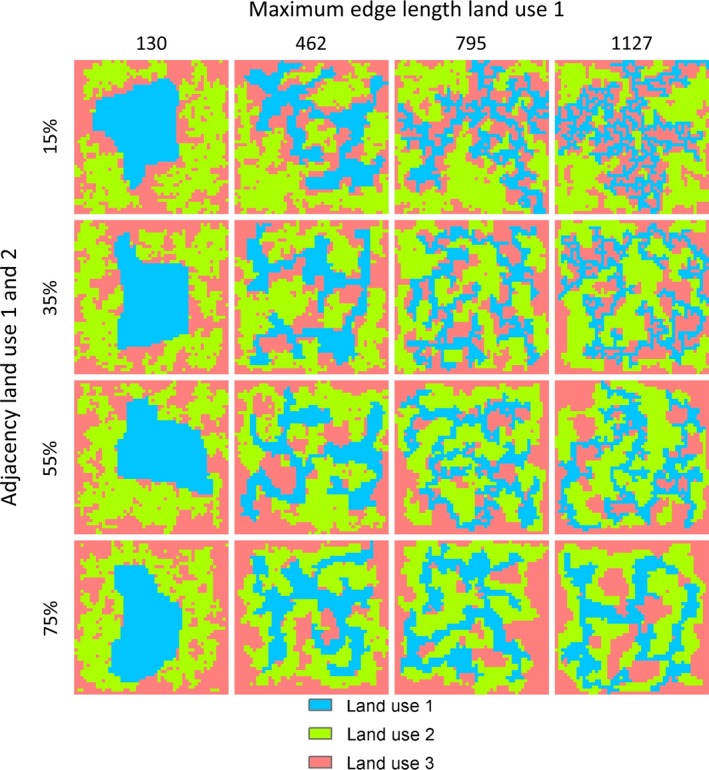
Landscape *series 2* generated by Landscape Generator. Landscapes in this series have three land‐use categories, various maximum edge lengths of land use 1 and different proportions of the adjacency between land use 1 and 2. For a detailed overview of the input settings, see Table 2.

LG successfully generated two example landscape series that could be used in landscape ecological simulation studies (Figs. 5, 6). In *series 1* (Fig. 5), we observe that the size of the largest patch increases with an increasing largest patch level. Similarly, the number of patches in each landscape corresponds to the number of patches indicated in the input settings. Note that diagonally connected patches are considered separate patches in LG (i.e., four‐neighbor criterion). As we allowed a one percent point maximum deviation from the AREA target value, the size of the smaller patches in landscapes with largest patch level 4 is quite variable (e.g., a target value of 2% AREA, can range between 1% and 3%).

In *series 2* (Fig. 6), in accordance to the input settings, an increasing adjacency between LU1 and LU2 can be observed with an increasing edge length of the patch of LU1. However, for the generated landscapes with PERIM = 55% and ECON = 795 or 1127 as well as those with PERIM = 75% and ECON = 462, 795 or 1127, not much difference can be observed between the edge lengths of the LU1 patch. Apparently, to achieve the other input settings for these landscapes, the actual edge length of the LU1 patch was below the permissible maximum edge length. All 16 landscapes for *series 1* were generated in 0:23 h. For *series 2*, 14 out of 16 landscapes were generated in 2:43 h. The remaining two landscapes were later generated in 3:37 h.

## Discussion

In this study, we demonstrate how LG can be used to generate neutral landscapes as input for landscape ecological simulations. Whereas traditional neutral landscape models implement a relatively small set of landscape‐level pattern metrics, LG implements a more complete set of class‐ and patch‐level pattern metrics, which gives LG users more control over the final landscape pattern and allows them to generate a wider variety of patterns. This enables scientists to test hypothesis on the effect of landscape patterns on spatial ecological processes that were previously unfeasible. As we here demonstrated, LG was capable of recreating landscape patterns from real landscapes with a fairly high level of accuracy and precision. The input settings in LG thus encompass the main class‐, and patch‐level pattern variables necessary to describe real landscapes. Most previous neutral landscape models are based on stochastic or probabilistic processes of which probabilities can be specified to influence the pattern of the generated landscape. With LG, however, a spatial optimization algorithm is used to generate a landscape based on user‐defined target values of pattern variables. Although the algorithm used in LG is not entirely deterministic and several generated landscapes with matching input settings will not be identical, the variability between landscape pattern variables from simulated landscapes with identical input settings was small in most cases. By increasing the number of pattern variables in the input settings, users of LG can further restrict the variability in output landscapes. This, however, usually comes at the cost of longer calculation times or a higher probability of an unfeasible landscape configuration. With the current version of LG, users can specify all the input settings in simple text files and landscapes can be generated simultaneously on all processor cores.

Many of the studies that make use of generated landscape maps to study the effect of landscape patterns on ecological processes will use the rasters directly as input variables to simulation models (e.g., Wiegand et al. 1999; Flather and Bevers 2002; Steiner and Köhler 2003; Bruggeman et al. 2010). For instance, the generated example landscape series in this study (Figs. 5, 6) allows simulating ecological processes in landscapes with varying composition of land uses, number of patches, patch sizes, edge lengths or land‐use class adjacencies. The ecological processes can be simulated with software such as SimAdapt (Rebaudo et al. 2013) with which genetic patterns resulting from landscape permeability can be simulated. Another example is the “stochastic movement simulator” presented in Palmer et al. (2011) with which habitat connectivity can be assessed in a landscape. Both software packages allow users to import categorical landscape maps.

Despite the advantages of LG mentioned above, there are still some potential improvements to LG that would further increase its usability. First, some adjustments could be made to the currently implemented pattern variables, in order to give users even more control over the generated landscape. For instance, it would be an improvement if users were able to specify an absolute target value for edge length, opposed to the currently implemented maximum edge length. Also, changing patch edge contrast (ECON; Table 1) from a patch‐level to a class‐level target value (i.e., mean patch edge contrast; ECON_MN) would allow users to specify the overall adjacency between two land‐use classes, opposed to having to specify this adjacency for each patch separately. However, such adjustments could also lead to objectives that become so restrictive that LG is unable to find the optimal landscape patterns. Second, the speed at which LG generates landscapes could be improved. Whereas small landscapes of 20 × 20 raster cells are generated in few minutes (Slager and de Vries 2013), the larger landscapes of 50 × 50 raster cells generated in this study took multiple hours to complete. Calculation time in LG thus increases exponentially with increasing size of the landscape. Implementing more efficient spatial optimization algorithms may speed up the code significantly. For instance, Huang et al. (2012) developed a spatial multiobjective optimization algorithm based on an “artificial immune system”. These authors demonstrated their algorithm on a landscape of 389 × 337 cells with four land‐use categories and found optimal solutions for two spatial objectives in approximately 1:20 h. In Huang et al. (2012), the spatial objectives were planning objectives (i.e., spatial suitability and compactness), but perhaps such an algorithm could also be implemented to optimize the pattern objectives in LG. Third, to further increase the ability of LG to recreate landscape patterns from real landscapes, several additional pattern variables should be implemented as objective functions in LG. One of these variables is, for instance, the nearest neighbor distance, which is important for the description of landscape patterns (Cushman et al. 2008), and is one of the landscape metrics that differed most between our real and recreated landscapes (Fig. 4). Another example of a potentially useful pattern variable is a metric for largest patch size. Although the size and perimeter of patches can be indicated in the current version of LG, one cannot indicate that all other patches should be smaller. As we only indicated the size and perimeter of three large patches in our recreated landscapes, larger patches or patches with longer perimeters could have been formed. This is a likely cause for the lower accuracy for metrics quantifying patch shape complexity (SHAPE_MN) and largest patch dominance (LPI; Cushman et al. 2008). An alternative to implementing a largest patch input parameter is to indicate the patch sizes of all patches in a landscape, as we have done in landscape *series 1*. With the modular setup of the LG, new pattern variables can be implemented with relative ease.

Here, we show how a series of landscapes can be generated in which each landscape is independent of the other landscapes. However, if the process under investigation is actually affected by the changes between landscapes (e.g., habitat loss or urban expansion) a time series of landscapes should be generated in which each new landscape is based on the previous landscape. Based on traditional neutral landscape models, Cambui et al. (2015) developed software that automatically creates such time series of binary landscapes. LG can also be used to generate similar time series of landscapes undergoing a certain change. For each raster cell in the input raster, one can indicate whether it can or cannot be swapped with raster cells of other land‐use categories. Thus, by fixing the raster cells in certain patches, new landscape configurations can be generated around the fixed patches. This process can then be repeated to create a time series of landscapes undergoing change.

## Conflict of Interest

None declared.

## Data Accessibility

The LG program together with a user's manual and example files can be downloaded from www.lg.ethz.ch.

## Supporting information


**Appendix S1.** Input settings for Landscape Generator (LG; Slager and de Vries (2013) Landscape generator: method to generate landscape configurations for spatial plan‐making. Computers Environment and Urban Systems, 39, 1–11) to re‐create the land‐use pattern in the Canton of Valais, Switzerland.
