# Supplementary material for: An improved neutral landscape model for recreating real landscapes and generating landscape series for spatial ecological simulations
Source: Ecol Evol. 2016 May 9;6(11):3808–21. doi: 10.1002/ece3.2145 (PMC4865471; doi:10.1002/ece3.2145)
Supplement: Supplementary file 1 — Appendix S1. Input settings for Landscape Generator (LG; Slager and de Vries (2013) Landscape generator: method to generate landscape configurations for spatial plan‐making. Computers Environment and Urban Systems, 39, 1–11) to re‐create the land‐use pattern in the Canton of Valais, Switzerland. [file ECE3-6-3808-s001.docx]

**Appendix S1.**

Input settings for Landscape Generator (LG; Slager, C.T.J. & de Vries, B. (2013) Landscape generator: method to generate landscape configurations for spatial plan-making. Computers Environment and Urban Systems, 39, 1-11) to re-create the land-use pattern in the Canton of Valais, Switzerland. The input settings are derived from landscape metrics calculated for land-use patterns in different slope classes: flat = 0-10%; moderate = 11-40%; steep ≥ 41%. Input for LG consisted of the following settings for the land-use categories settlement, agriculture and forest: percentage of total area (PLAND), number of patches (NP), total edge (TE), the sizes of the three largest patches as percentage of the area occupied by the respective land-use (AREA) and the perimeter of those three patches (PERIM).

| **Input Landscape Generator** | **Flat terrain slope** | **Moderate terrain slope** | **Steep terrain slope** |
| --- | --- | --- | --- |
| PLAND settlement (% area) | 33.67 | 12.55 | 2.12 |
| PLAND agriculture (% area) | 56.44 | 53.86 | 17.68 |
| PLAND forest (% area) | 9.89 | 33.59 | 80.20 |
| NP settlement | 116 | 157 | 82 |
| NP agriculture | 122 | 337 | 322 |
| NP forest | 72 | 356 | 243 |
| TE settlement (m) | 98700 | 93900 | 36900 |
| TE agriculture (m) | 127500 | 302400 | 220100 |
| TE forest (m) | 41200 | 231700 | 453900 |
| AREA largest settlement patch (% area) | 11 | 8 | 4 |
| PERIM largest settlement patch (m) | 6400 | 3600 | 1000 |
| AREA 2nd largest settlement patch (% area) | 6 | 6 | 4 |
| PERIM 2nd largest settlement patch (m) | 4200 | 3000 | 1000 |
| AREA 3rd largest settlement patch (% area) | 4 | 4 | 4 |
| PERIM 3rd largest settlement patch (m) | 3400 | 2500 | 1000 |
| AREA largest agriculture patch (% area) | 4 | 6 | 10 |
| PERIM largest agriculture patch (m) | 4800 | 8600 | 8100 |
| AREA 2nd largest agriculture patch (% area) | 3 | 5 | 3 |
| PERIM 2nd largest agriculture patch (m) | 3700 | 7100 | 3300 |
| AREA 3rd largest agriculture patch (% area) | 3 | 4 | * |
| PERIM 3rd largest agriculture patch (m) | 3700 | 6200 |  |
| AREA largest forest patch (% area) | 5 | 3 | 12 |
| PERIM largest forest patch (m) | 1400 | 4000 | 32900 |
| AREA 2nd largest forest patch (% area) | 4 | 3 | 3 |
| PERIM 2nd largest forest patch (m) | 1300 | 3600 | 11900 |
| AREA 3rd largest forest patch (% area) | 3 | * | 3 |
| PERIM 3rd largest forest patch (m) | 1200 |  | 10700 |
| * Largest patches were only considered if AREA was larger than 2 % | | |  |
